# Analysis of the clinical characteristics of 77 COVID-19 deaths

**DOI:** 10.1038/s41598-020-73136-7

**Published:** 2020-10-02

**Authors:** Kaige Wang, Zhixin Qiu, Jiasheng Liu, Tao Fan, Chunrong Liu, Panwen Tian, Ye Wang, Zhong Ni, Shumin Zhang, Jianfei Luo, Dan Liu, Weimin Li

**Affiliations:** 1 Department of Pulmonary and Critical Care Medicine, West China Hospital, Sichuan University, Chengdu, People’s Republic of China; 2 Department of Gastrointestinal Surgery, Renmin Hospital of Wuhan Uiniversity, Wuhan, People’s Republic of China; 3 Pneumology Group, Department of Integrated Traditional Chinese and Western Medicine, West China Hospital, Sichuan University, Chengdu, People’s Republic of China; 4 Chinese Evidence-Based Medicine Center, West China Hospital, Sichuan University, Chengdu, People’s Republic of China; 5 State Key Laboratory of Virology, Wuhan Institute of Virology, Chinese Academy of Science (CAS), Wuhan, People’s Republic of China

**Keywords:** Diseases, Medical research

## Abstract

The COVID-19 outbreak is becoming a public health emergency. Data are limited on the clinical characteristics and causes of death. A retrospective analysis of COVID-19 deaths were performed for patients’ clinical characteristics, laboratory results, and causes of death. In total, 56 patients (72.7%) of the decedents (male–female ratio 51:26, mean age 71 ± 13, mean survival time 17.4 ± 8.4 days) had comorbidities. Acute respiratory failure (ARF) and sepsis were the main causes of death. Increases in C-reactive protein (CRP), lactate dehydrogenase (LDH), D-dimer and lactic acid and decreases in lymphocytes were common laboratory results. Intergroup analysis showed that (1) most female decedents had cough and diabetes. (2) The proportion of young- and middle-aged deaths was higher than elderly deaths for males, while elderly decedents were more prone to myocardial injury and elevated CRP. (3) CRP and LDH increased and cluster of differentiation (CD) 4+ and CD8+ cells decreased significantly in patients with hypertension. The majority of COVID-19 decedents are male, especially elderly people with comorbidities. The main causes of death are ARF and sepsis. Most female decedents have cough and diabetes. Myocardial injury is common in elderly decedents. Patients with hypertension are prone to an increased inflammatory index, tissue hypoxia and cellular immune injury.

## Introduction

In December 2019, the first cases of a pneumonia of unknown origin were identified in Wuhan, Hubei, China^1^. On January 7, 2020, severe acute respiratory syndrome coronavirus 2 (SARS-CoV-2) was isolated by Chinese scientists from patients with the disease^2^, which was officially named coronavirus disease 2019 (COVID-19) by the World Health Organization (WHO) in February 2020. Several articles confirmed that the virus spread from person to person both in hospitals settings and among families^3^. The clinical spectrum of SARS-CoV-2 infection encompasses asymptomatic infection, mild upper respiratory tract infection, and severe viral pneumonia with respiratory failure and even death. A number of published case studies made summaries of all patients infected with SARS-CoV-2^4,5^, and some death cases were included^6^. However, no clear descriptions or analyses of the clinical characteristics of patients who died of COVID-19 and the causes of death are available. Hence, the detailed clinical data of 77 COVID-19 deaths in the RHWU East Branch were included in this study, and a thorough analysis of their clinical characteristics and laboratory test results, as well as comparisons among different subgroups, was performed.

## Method

### Study design

The clinical and laboratory data of 77 patients who died of COVID-19 in the RHWU East Branch from February 1, 2020, to March 7, 2020, were collected. The hospital is a designated hospital for the treatment of severe and critical COVID-19 patients in Wuhan, where all patients are diagnosed with COVID-19 according to the WHO interim guidance. Among 79 deaths during this period, two died immediately after admission and were excluded because of a lack of clinical data. Therefore, the clinical and laboratory data of 77 deaths were collected and analysed in this study after the approval of the RHWU Research Ethics Committee (WDRY2020-K068). Informed consent was obtained from Legally Authorized Representative or next to kin of the deceased patients.

### Data collection

The epidemiologic, demographic, clinical characteristics, laboratory test and treatment plan data were extracted from electronic medical records in accordance with the modified version of the WHO/International Severe Acute Respiratory and Emerging Infection Consortium (ISARIC) case record form for severe acute respiratory infections. All data were reviewed by two doctors, and then, a third researcher (LD) determined whether there was a difference in interpretation between the two reviewers.

### Laboratory test process

The laboratory confirmation method of SARS-CoV-2 infection has been explained in other articles. Real-time RT-PCR assays have been used to detect SARS-CoV-2 in respiratory specimens of patients in other designated hospitals for COVID-19 treatment. Patients with positive results on two assays and pulmonary infection detected by chest CT were confirmed to have COVID-19 and admitted to the RHWU East Branch. After admission, all patients’ respiratory specimens were tested again for further diagnosis of SARS-CoV-2 infection. Laboratory tests such as ECG and arterial blood gas analysis and determination of routine complete blood count (CBC), coagulation function, serum biochemical indexes (incl. liver and kidney functions, creatine kinase and lactate dehydrogenase), myocardial enzymes, brain natriuretic peptide, CRP and procalcitonin (PCT) were carried out. In addition, all patients received a chest X-ray or CT scan at least once, and the examination frequency was determined by their doctors.

### Definitions

Young and middle-aged patients refer to patients aged 18–65 years old, and elderly patients refer to patients over 65 years old. An axillary temperature above 37.6 °C (99.7°F) is considered a fever. The diagnosis of sepsis and septic shock is based on the Third International Consensus Definitions for Sepsis and Septic Shock (Sepsis-3) issued in 2016^7^. The diagnosis of acute kidney injury (AKI) is based on the Kidney Disease Improving Global Outcomes (KDIGO) Clinical Practice Guideline for AKI^8^. Acute myocardial infarction (AMI) is diagnosed by an increase in the serum level of cardiac biomarkers (such as high-sensitivity cardiac troponin I) or new abnormalities found in ECG and echocardiography. According to the *Diagnosis and Treatment Plan for Coronavirus Disease 2019* (Tentative Sixth Edition)^9^, the severity of COVID-19 is defined as follows: Typical cases—Patients have fever, respiratory tract symptoms, etc. and pneumonia manifestations can be seen in imaging. Severe cases have any of the following—1. Respiratory distress, RR ≥ 30 breaths/min; 2. oxygen saturation ≤ 93% at rest; or 3. arterial partial pressure of oxygen (PaO_2_)/oxygen concentration (FiO_2_) ≤ 300 mmHg (1 mmHg = 0.133 kPa). Critical cases have any of the following—1. Respiratory failure requiring mechanical ventilation; 2. shock; or 3. other organ failure complications that require monitoring and treatment in the ICU. Cases with close contact at home refer to the diagnosis of COVID-19 in two or more family members or people who had close contact within 14 days. Short-term survival refers to the survival time from disease onset to death ≤ 14 days.

### Statistical method

Data were analysed by SPSS version 19.0. Normally distributed measurements are expressed as means ± SD. A t-test was used to compare the means of two groups, and ANOVA was used to compare the means of more than two groups. Non-normally distributed measurements are expressed as the median and were analysed by the Wilcoxon rank-sum test. A chi-square, denoted by χ^2^, test was used to analyse enumeration data, and a value of *P* < 0.05 was considered statistically significant.

### Informed consent

For this retrospective study, written informed consent was provided by the subjects’ legally authorized ext of kin. The subjects’ legally authorized representatives or next of kin provided informed consent for the publication of the manuscript and to publish the data in an online open access publication.

## Results

### Analysis of clinical characteristics

From January 31, 2020, to March 7, 2020, a total of 1,179 COVID-19 patients were admitted to the EHWU East Branch and 77 (incl. 51 males and 26 females) of those patients died. The mean age at death was 71 ± 13 years old, the number of COVID-19 deaths in males was higher than that in females (66.2% vs 33.8%), and the mean age at death for males was lower than that in females (69 ± 15 vs 75 ± 10). All patients were permanent residents of Wuhan. Among them, 81.8% (63 patients) of infections were caused by mass gatherings, and most patients had fever and dyspnoea. A total of 56 patients (72.8%) had underlying diseases, and hypertension (33, 58.9%) was the most common, followed by diabetes (18, 32.1%), heart disease (18, 32.1%), and chronic lung disease (8, 14.3%); 33 patients (43.0%) had a history of smoking (Table 1). According to the assessment results within 24 h after admission, 71 were critical cases, 4 were severe cases and 2 were typical cases. Among the typical patients, one patient died of cerebral haemorrhage after kidney transplantation, while the other suffered from massive cerebral infarction and died of cerebral herniation.

**Table 1:** Clinical characteristics of COVID-19 deaths.

Clinical characteristics	All cases (N = 77) , n(%), mean(SD)
**Gender**	
Male	51 (66.2)
Female	26 (33.8)
**Age at death, years**	71 (13)
Male	69 (15)
Female	75 (10)
**Mean survival time (from disease onset to death), days**	17.4 (8.4)
**Permanent residents of Wuhan**	77 (100.0)
**Mass gathering**	63 (81.8)
**Initial symptoms**	
Fever	63 (81.8)
Cough	15 (19.5)
Dyspnea	25 (32.5)
Fatigue	12 (15.6)
Abdominal pain, diarrhea	3 (3.9)
Neurological symptoms	4 (5.2)
**Comorbidities**	
**Any of comorbidities**	56 (72.7)
Hypertension	33 (58.9)
Heart disease	18 (32.1)
Diabetes	18 (32.1)
Chronic lung disease (Chronic obstructive pulmonary disease, Asthma, Bronchiectasis)	8 (14.3)
Cerebrovascular disease	7 (12.5)
Nephropathy	7 (13.5)
Liver disease	4 (7.1)
Cancers	2 (3.6)
Surgery	1 (1.8)
Others (Rectal polyps, Gout, Gallstone, Pancreatitis)	4 (7.1)
**Smoking history, yes**	33 (42.9)
**Causes of death**	
Acute respiratory distress syndrome^a^	67 (87.0)
Sepsis^a^	14 (18.2)
Nervous system disease^a^	3 (3.9)
Heart disease^a^	2 (2.6)
Gastrointestinal bleeding^a^	2 (2.6)
Others (Pancreatitis, Uremia, Renal insufficiency)^a^	3 (3.9)
**Respiratory support**	
Invasive mechanical ventilation	7 (9.1)
Non-invasive ventilator	24 (31.2)
Oxygen mask	24 (31.2)
High-flow nasal cannula oxygen therapy	16 (20.8)
**Mean time from admission to endotracheal intubation, days**	6 (3.3)

^a^Analysis was done in the cases with any comorbidity, so the total number is 56 for all.

### Laboratory tests

The results of laboratory tests, including haemograms, T cell subsets, D-dimer levels, and biochemical and inflammatory marker levels within 24 h of readmission, are summarized in Table 2. *Klebsiella pneumoniae* was cultured from one blood specimen. The sputum culture results showed identified two case of *Pseudomonas aeruginosa*, one *Acinetobacter baumannii*, one *Staphylococcus aureus*, two *Candida albicans* and one *Aspergillus* among the patients.

**Table 2:** Initial laboratory analysis of COVID-19 deaths.

Initial laboratory analysis	N = 77, n(%), median(IQR)
**Complete blood count**	
White blood cell count; × 10^9^/L; normal range 3.5–9.5	11.305 (6.745, 15.123)
Increase	36 (46.8)
Decreased	9 (11.7)
Lymphocyte count; × 109/L; normal range > 1.1	0.54 (0.38, 0.86)
Decreased	67 (87.0)
Neutrophil count; × 10^9^/L; normal range 1.8–6.3	9.815 (5.555, 14.058)
Increase	50 (64.9)
Hemoglobin; g/L; normal range > 130	127 (111, 139)
Decreased	43 (55.8)
Mean corpuscular volume; fl; normal range 80–100	92.7 (87.6, 98.25)
Decreased	3 (3.9)
Platelet count; × 10^9^/L; normal range 125–300	151 (89, 208)
Decreased	25 (32.5)
**C-reactive protein; mg/L; normal range > 10**	94 (61.75, 151.9)
Increase	69 (89.6)
**Procalcitonin; ng/mL; normal range < 0.1**	0.35 (0.138, 1.330)
** > 0.1**	57 (74.0)
** > 0.5**	29 (37.7%)
**Myocardial markers**	
Creatine kinase-MB; ng/mL; normal range < 5	2.72 (1.69, 4.87)
Increase	14 (18.2)
Troponin; ng/mL; normal range < 0.04 ng/mL	0.13 (0.028, 0.682)
Increase	34 (44.2)
Brain natriuretic peptide; pg/mL; normal range < 900	847.9 (424.45, 1623)
Increase	26 (33.8)
**Blood biochemical analysis**	
Alanine aminotransferase; U/L; normal range < 50	25 (19, 52.5)
Increase	17 (22.1)
Aspartate aminotransferase; U/L; normal range < 40	41 (27.25, 71.5)
Increase	38 (49.4)
Creatinine; μmol/L; < 111	73 (58, 112)
Increase	19 (24.7)
Lactic dehydrogenase; U/L; normal range < 250	570 (476, 736.75)
Increase	68 (88.3)
Creatine kinase; U/L; normal range < 310	70 (45.5, 141.5)
Increase	8 (10.4)
**Coagulation**	
Prothrombin time; s; normal range 9–16	13.1 (12.4, 14.2)
Decrease	0 (0.0)
Activated partial prothrombin time; s; normal range 28–44	28.8 (26.95, 31.75)
Decrease	4 (5.2)
D-dimer; mg/L; normal range < 0.55	6.88 (2.84, 17.57)
Increase	59 (76.6)
**Cell immunity**^**a**^	
CD4+ cell count; /µL; normal range > 404	89 (126, 274)
** < **404/µL	52 (98.1)
** < **202/µL	32 (60.4)
CD8+ cell count, /µL; normal range > 220/µL	89 (44.25, 154.5)
Decrease	43 (81.1)
**Lactic acid in blood gas analysis; mmol/L; < 1.5**	2.5 (2.1, 3.7)
Increase	68 (88.3)

^a^The below analysis was done in the cases with the result of cell immunity, so the total number is 53 for all.

**Table 3:** Comparison of clinical characteristics among different subgroups.

Characteristics	All (N = 77)	Gender			Age at death			Survival time, days			Complicated with hypertension^d^		
		Male (n = 51)	Female (n = 26)	*P* value*	≤ 65 y (n = 24)	> 65y (n = 53)	*P* value*	< 14 days (n = 29)	≥ 14 days (n = 48)	*P* value*	Yes (n = 32)	No (n = 24)	*P* value*
**Age at death, mean(SD), years**	71 (13)	69 (15)	75 (10)	0.057^b^	NA	NA	NA	71 (11)	71 (14)	0.906^b^	74 (16)	74 (11)	0.962^b^
**Male**	51(66.2)	NA	NA	NA	20 (83.3)	31 (58.5)	**0.033**	22 (75.9)	29 (60.4)	0.165	24 (75.0)	14 (58.3)	0.186
**Mean survival time (from disease onset to death) (SD), days**	17.4 (8.4)	16.9 (7.0)	18.5 (8.6)	0.418 ^b^	17.0 (9.3)	17.6 (7.2)	0.774^b^	NA	NA	NA	17.1 (9.4)	17.8 (8.1)	0.773^b^
**Initial symptoms**													
Fever	63 (81.8)	40 (78.4)	23 (88.5)	0.360^a^	20 (83.3)	43 (81.1)	1.000^a^	24 (82.8)	39 (81.3)	0.868	27 (84.4)	17 (70.8)	0.222
Cough	15 (19.5)	6 (11.8)	9 (34.6)	**0.017**	5 (20.8)	10 (18.9)	1.000^a^	7 (24.1)	8 (16.7)	0.423	9 (28.1)	6 (25.0)	0.794
Dyspnea	25 (32.5)	14 (27.5)	11 (42.3)	0.188	7 (29.2)	18 (34.0)	0.677	9 (31.0)	16 (33.3)	0.835	13 (40.6)	5 (20.8)	0.117
Fatigue	12 (15.6)	10 (19.6)	2 (7.7)	0.318^a^	1 (4.2)	11 (20.8)	0.091^a^	3 (10.3)	9 (18.8)	0.518^a^	3 (9.4)	4 (16.7)	0.447^a^
Abdominal pain, diarrhea	3 (3.9)	3 (5.9)	0 (0.0)	0.547^a^	1 (4.2)	2 (3.8)	1.000^a^	2 (6.9)	1 (2.1)	0.553^a^	1 (3.1)	1 (4.2)	1.000^a^
Neurological symptoms	4 (5.2)	4 (7.8)	0 (0.0)	0.294^a^	1 (4.2)	3 (5.7)	1.000^a^	3 (10.3)	1 (2.1)	0.147^a^	1 (3.1)	1 (4.2)	1.000^a^
**Comorbidities**													
Any of comorbidities	56 (72.7)	38 (74.5)	18 (69.2)	0.623	15 (62.5)	41 (77.4)	0.175	21 (72.4)	35 (72.9)	0.962	NA	NA	NA
Hypertension^c^	33 (58.9)	24 (63.2)	9 (50.0)	0.350	8 (53.3)	25 (61.0)	0.607	12 (57.1)	21 (60.0)	0.833	NA	NA	NA
Heart disease^c^	18 (32.1)	12 (31.6)	6 (33.3)	0.896	4 (26.7)	14 (34.1)	0.751^a^	9 (42.9)	9 (25.7)	0.184	NA	NA	NA
Diabetes^c^	18 (32.1)	7 (18.4)	11 (61.1)	**0.001**	3 (20.0)	15 (36.6)	0.338^a^	4 (19.0)	14 (40.0)	0.104	NA	NA	NA
Chronic lung disease^c^	8 (14.3)	5 (13.2)	3 (16.7)	0.703^a^	4 (26.7)	4 (9.8)	0.190^a^	1 (4.8)	7 (20.0)	0.235^a^	NA	NA	NA
Cerebrovascular disease^c^	7 (12.5)	5 (13.2)	2 (11.1)	1.000^a^	0 ((0.0))	7 (17.1)	0.171^a^	3 (14.3)	4 (11.4)	1.000^a^	NA	NA	NA
Nephropathy^c^	7 (13.5)	4 (10.5)	3 (16.7)	0.669 ^a^	2 (13.3)	5 (12.2)	1.000^a^	2 (9.5)	5 (14.3)	0.700^a^	NA	NA	NA
Liver disease^c^	4 (7.1)	2 (5.3)	2 (11.1)	0.587 ^a^	1 (6.7)	3 (7.3)	1.000^a^	1 (4.8)	3 (8.6)	1.000^a^	NA	NA	NA
Cancers^c^	2 (3.6)	2 (5.3)	0 (0.0)	1.000 ^a^	0 (0.0)	2 (4.9)	1.000^a^	2 (9.5)	0 (0.0)	0.136^a^	NA	NA	NA
Surgery^c^	1 (1.8)	1 (2.6)	0 (0.0)	1.000 ^a^	1 (6.7)	0 ((0.0))	0.268^a^	0 (0.0)	1 (2.9)	1.000^a^	NA	NA	NA
Others^c^	4 (7.1)	3 (7.9)	1 (5.6)	1.000 ^a^	1 (6.7)	3 (7.3)	1.000^a^	3 (14.3)	1 (2.9)	0.143^a^	NA	NA	NA
**Causes of death**													
ARF	67 (87.0)	43 (84.3)	24 (92.3)	0.480^a^	19 (79.2)	48 (90.6)	0.270^a^	23 (79.3)	43 (89.6)	0.314^a^	29 (90.6)	18 (75.0)	0.151^a^
Sepsis	14 (18.2)	12 (23.5)	2 (7.7)	0.122 ^a^	4 (16.7)	8 (15.1)	1.000^a^	8 (27.6)	6 (12.5)	0.096	8 (27.6)	4 (22.2)	0.452
Nervous system disease	3 (3.9)	2 (3.9)	1(3.8)	1.000 ^a^	2 (8.3)	1 (1.9)	0.228^a^	1 (3.4)	2 (4.2)	1.000^a^	0 (0.0)	3 (12.5)	0.073^a^
Heart disease	2 (2.6)	2 (3.9)	0 (0.0)	0.547^a^	0 (0.0)	2 (3.9)	1.000^a^	2 (6.9)	0 (0.0)	0.139^a^	1 (3.4)	1 (4.2)	1.000^a^
Gastrointestinal bleeding	2 (2.6)	2 (3.9)	0 (0.0)	0.547^a^	0 (0.0)	2 (3.9)	1.000^a^	1 (3.4)	1 (2.1)	1.000^a^	1 (3.4)	1 (4.2)	1.000^a^
Others	3 (3.9)	2 (3.9)	1 (3.8)	1.000 ^a^	2 (8.3)	1 (1.9)	0.228^a^	2 (6.9)	1 (2.1)	0.553^a^	0 (0.0)	3 (12.5)	0.073^a^
**Mean time from admission to endotracheal intubation(SD), days**	6(3.3)	5(3.8)	8(1.4)		5(4.6)	7(1.5)		6(1.3)	9(4.4)		8(3.9)	1(1.2)	

*NA* not applicated.

*Chi-square test is used except for special denote.

^a^Fisher’s exact test.

^b^T test.

^c^Analysis was done in the cases with any comorbidity, so the total number is 56 for all cases; 38 for male and 18 for female among the gender subgroup; 15 for cases aged at 65 and below and 41 for cases aged beyond 65; 21 for cases with dead time less than 14 days and below and 35 for cases with dead time no less than 14.

^d^The patients including in this analysis was with at least one of comorbidities, so the total number of this analysis was 56 (32 for cases with hypertension, 24 for cases without hypertension).

### Imaging examination

All 77 patients underwent imaging examination (chest X-ray or CT) periodically from disease onset to death. Diffuse lesions (multiple ground-glass opacities likely due to exudate infiltration) in both lungs were found in the early stage and developed into large areas of bilateral lung consolidation, along with bronchiectasis and “white lungs” (asbestosis-like), in the later stage. Spontaneous pneumothorax and subcutaneous and mediastinal emphysema occurred in 2 patients who did not receive mechanical ventilation.

### Treatment plans and causes of death

Early treatment with oseltamivir was given to 58 patients. After admission, 75 patients (97.4%) received antiviral treatment with arbidol, 2 patients received lopinavir/ritonavir, and all patients received antibiotic treatment. Regarding respiratory support, 31 patients (40.3%) received mechanical ventilation, and 1 patient received extracorporeal membrane oxygenation (ECMO). ARF (67, 87.0%) was the main cause of death, followed by sepsis (14, 18.2%), neurological diseases (3, 3.9%), heart disease (2, 2.6%) and gastrointestinal bleeding (2, 2.6%). The mean survival time (from disease onset to death) was 17.4 ± 8.4 days. Only 7 patients (9.1%) underwent endotracheal intubation, and the mean time from admission to endotracheal intubation was 6 days. (Table 1).

### Intergroup analysis

**Figure 1:**
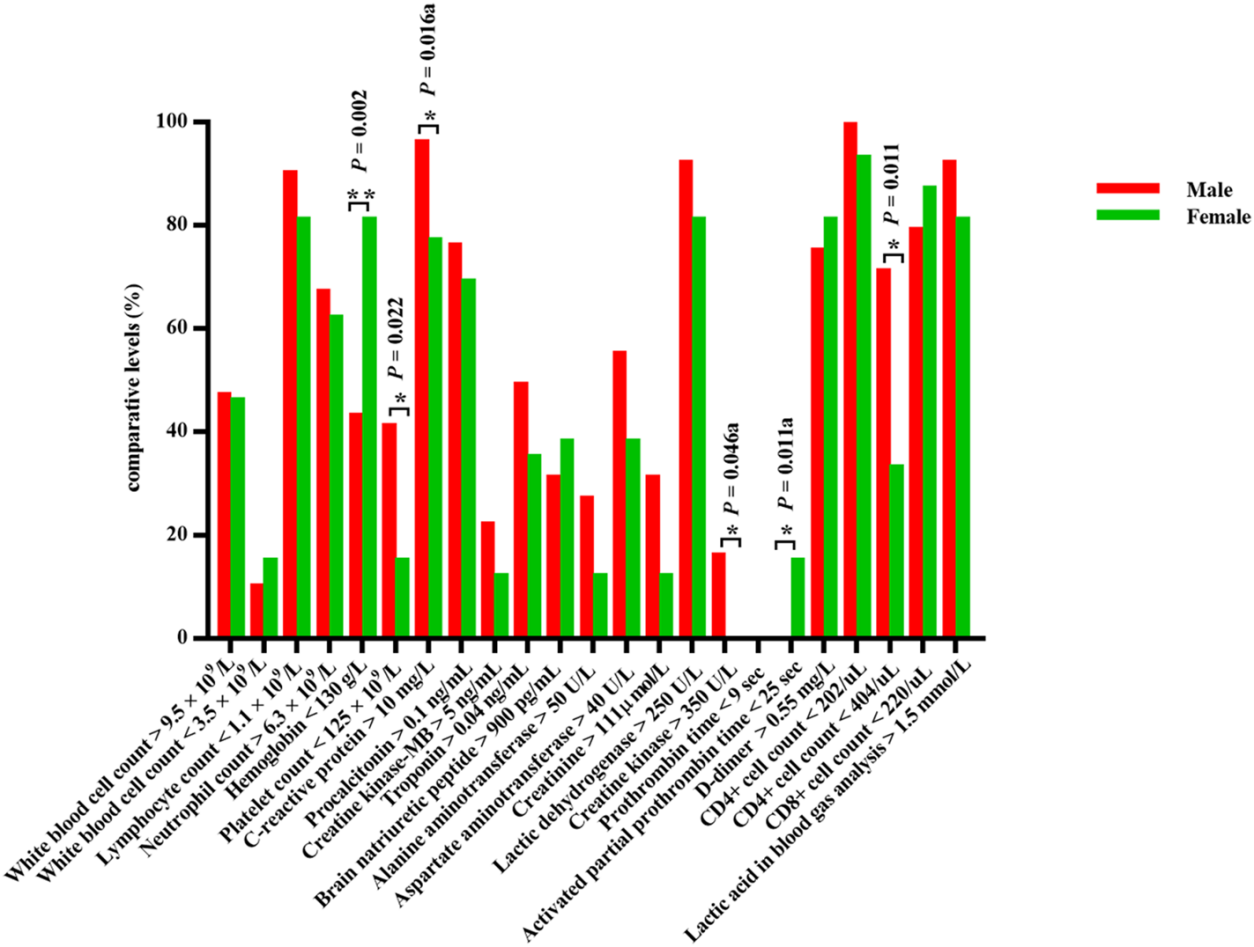
Comparison of clinical characteristics of COVID-19 patients with different gender.

**Figure 2:**
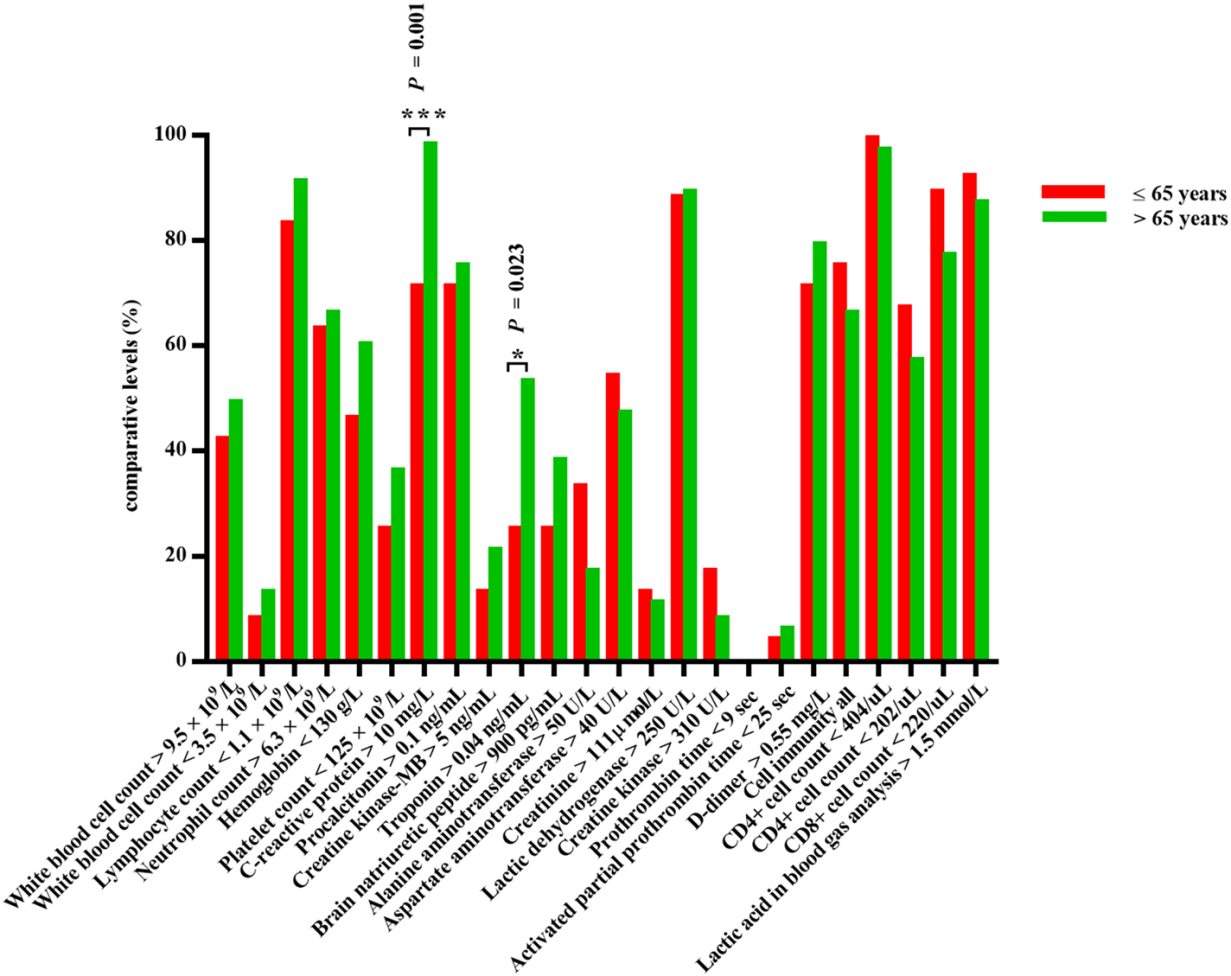
Comparison of clinical characteristics of COVID-19 patients with different age.

**Figure 3:**
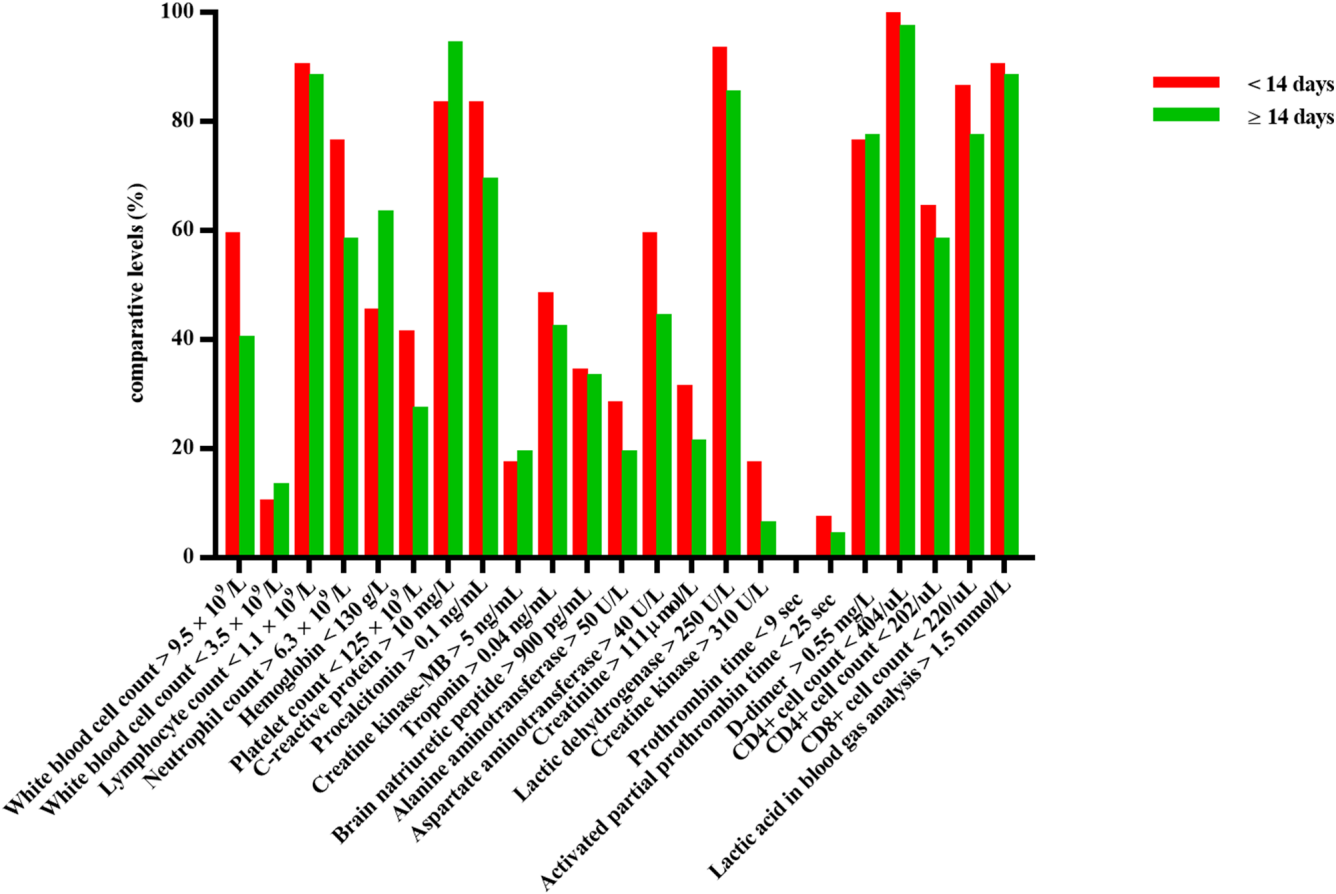
Comparison of clinical characteristics of COVID-19 patients with different survival time.

**Figure 4:**
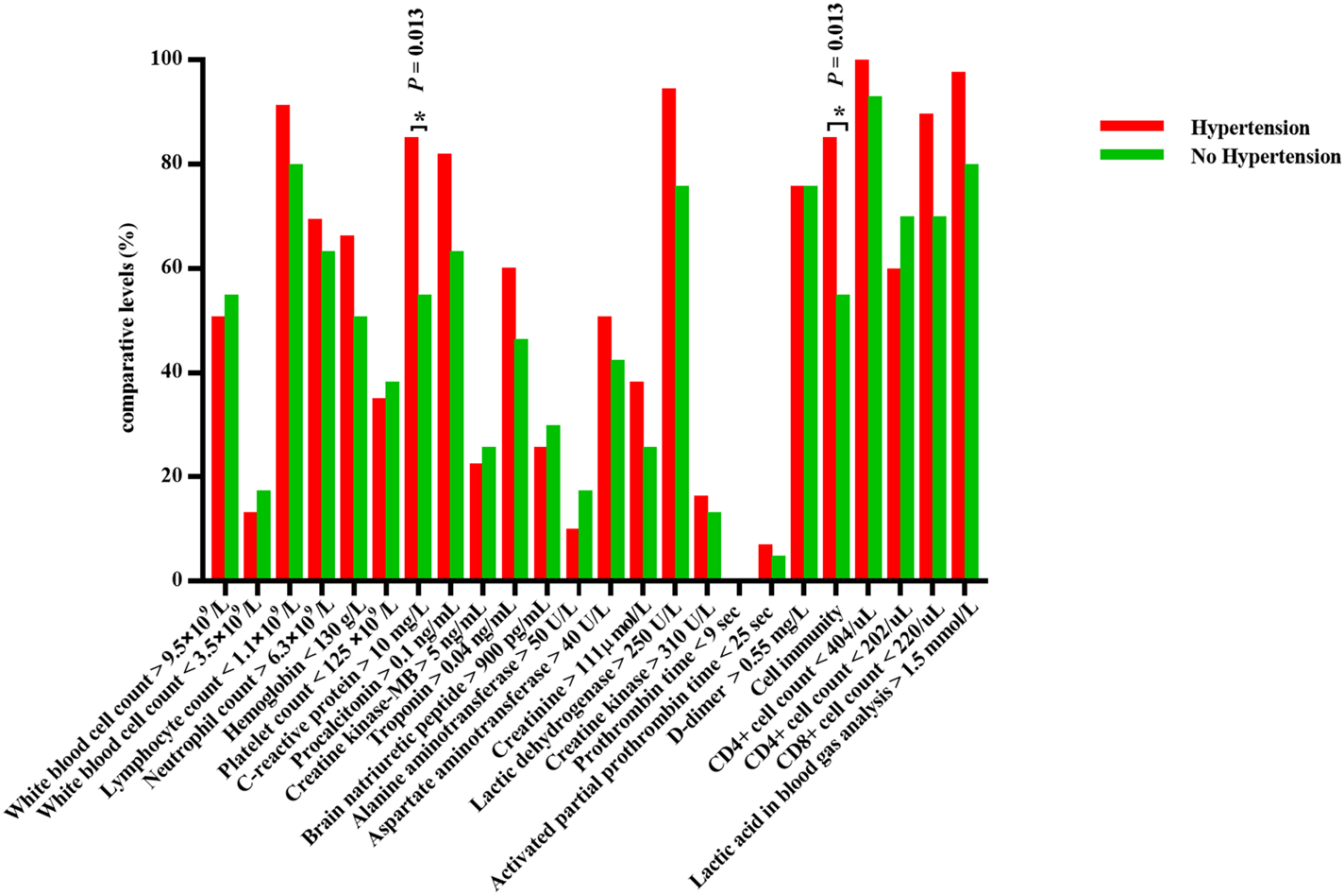
Comparison of clinical characteristics of COVID-19 patients with hypertension and without hypertension.

In this study, intergroup analysis was carried out based on a series of baseline characteristics, including gender, age, survival time and whether comorbid with hypertension, and the results were as follows: (1) Among the initial symptoms of patients who died, females were more prone to cough and dyspnoea (especially in the early stage) , and more likely to have diabetes, while the incidence of sepsis in males seemed to be higher than that in females. In addition, anaemia was more common in females, while elevated levels of CRP, creatinine and creatine kinase and decreased levels of platelets and CD4+ cells were more common in males (Fig. 1). (2) The majority of young and middle-aged deaths (≤ 65 years old) were males. Elderly patients seemed more prone to fatigue and myocardial injury, while young and middle-aged patients were more likely to have elevated levels of alanine aminotransferase and creatine kinase (Fig. 2). (3) The majority of short-term survival cases were males. In the short-term survival group, symptoms pertaining to the abdomen and nervous system were more likely to be found in the early stage, and the proportion of patients who had heart disease rather than diabetes or chronic lung disease was higher. The incidence of sepsis was higher in this subgroup, and laboratory tests were characterized by decreased platelets and elevated creatine kinase (Fig. 3). (4) Dyspnoea was more common in deaths complicated with hypertension, while fatigue and neurological symptoms were more common in deaths without hypertension. Compared with deaths without hypertension, CRP and LDH increased, and CD4+ cells and CD8+ cells decreased significantly in deaths complicated with hypertension (Fig. 4; Table 3).

### Case presentation

**Figure 5:**
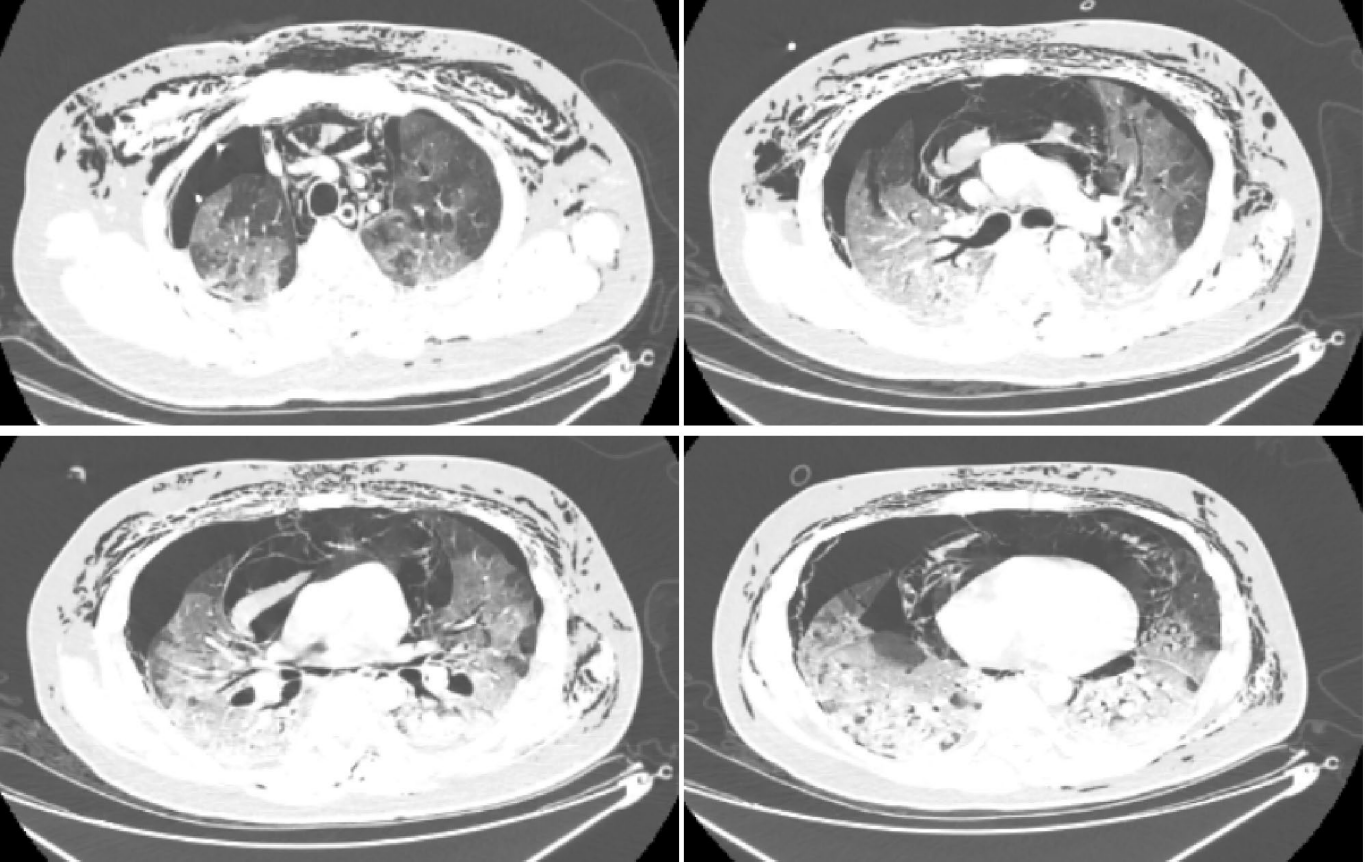
A 31-year-old male patient rapidly progressed to ARDS after onset and developed bilateral spontaneous pneumothorax.

A 31-year-old male patient, previously healthy, was admitted to the hospital on February 5, 2020, due to “fever for 13 days and dyspnoea for 2 days”. Before onset, he had been exposed to a COVID-19 patient. Oral administration of “oseltamivir 75 mg bid and moxifloxacin 0.5 g qd" was given; however, the effect was poor. The patient had two positive SARS-CoV-2 nucleic acid assays from throat swab specimens conducted by another hospital, and he was transferred to our hospital. Physical examination showed shortness of breath (28 breaths/min), oxygen saturation (by fingertip pulse oximeter) of 75%, and subcutaneous crepitus in the anterior chest along with reduced breath sounds in both lungs. The laboratory values were: leukocyte count: 15.5 × 10^9^/L; neutrophil count: 14.45 × 10^9^/L; lymphocyte count: 0.4 × 10^9^/L; CRP: 137.2 mg/L; PCT: 0.048 ng/L; creatine kinase: 473 U/L, LDH: 894 U/L, Alanine aminotransferase: 40 U/L, Aspartate aminotransferase: 30 U/L; CD4+ cells: 125/µL, and CD8+ cells: 160/µL; arterial blood gas analysis (oxygen mask at a flow rate of 8 L/min) showed pH: 7.48; PO_2_: 45 mmH_2_O; PCO_2_: 30 mmH_2_O; and Lac: 4 mmol/L. Chest CT showed bilateral pneumothorax and subcutaneous and mediastinal emphysema in the chest wall and neck and consolidation, exudative infiltrates and local compressive atelectasis in both lungs. After admission, the patient was given high-flow nasal oxygen therapy (flow rate: 60 L/min, oxygen concentration: 100%), closed chest drainage and an incision for subcutaneous emphysema and drainage of both lungs, and antiviral therapy with arbidol 200 mg tid (oral administration) + ribavirin 0.5 g bid (intravenous infusion). After the above treatment, dyspnoea was relieved and oxygen saturation reached 93%. Two days after admission, dyspnoea worsened, and the oxygenation index decreased further. The patient died on February 9 (Fig. 5).

## Discussion

This study includes a group of 77 COVID-19 deaths who were diagnosed and developed into critical cases in other hospitals from January to February 2020 and were then admitted to the temporary infection ICU of the RHWU East Branch. The clinical manifestations of this group are very similar to those of SARS-CoV. According to the literature, the shortest time from admission to ARDS is 2 days, and the mortality rate is 15–61.5%^1,6,10^. Recent studies have shown that nearly 70% of patients infected with SARS-CoV-2 are men^1,11^. In this study, men accounted for 66.2% of deaths, which is consistent with other studies. The majority of deaths were in elderly patients (53/77), which is consistent with the conclusion that risk of death increases with age^6^. The majority of both young- and middle-aged deaths and elderly deaths were in male patients, and the male proportion in the former group was comparatively higher than that of the latter. Female deaths were more likely to be complicated with diabetes. Approximately 81.8% of the patients were infected at mass gatherings with no exposure to the Huanan seafood market, which suggests that most of the deaths may be second-generation infected persons. This study shows that fever and respiratory, neurological and digestive symptoms were the common initial clinical manifestations of the patients who died, which is consistent with a previous study^1^. According to Cao Bin's analysis of the first-batch of 191 patients admitted to the ICU, 48% of COVID-19 patients had underlying diseases^6^ and that population in our study reached 72.8%. It can be seen that more deaths occur in patients who have underlying diseases. However, there is no relevant research on whether the mortality rate of patients with underlying diseases is higher than that of patients without underlying diseases or how various underlying diseases affect the progression to death. For the first time, a subgroup analysis based on hypertension was conducted in this study.

In terms of laboratory tests, 88% of the patients included in this study had lymphocytopenia, which is a prominent feature of patients with severe SARS-CoV infection because the targeted invasion of SARS-CoV particles can cause the destruction of lymphocytes by destroying their cytoplasmic components^12^. In addition, patients with severe MERS infection also commonly have lymphocytopenia due to lymphocyte apoptosis^13,14^. Most recent studies have confirmed that lymphocytes in patients with critical COVID-19 decrease significantly compared with lymphocyte counts in patients with mild disease. Some studies have reported that lymphocytopenia is a high-risk factor for death^6^. Others have also shown that the neutrophil-to-lymphocyte ratio (NLR) can be used as a risk factor for the early prediction of critical COVID-19 cases, namely, patients with age ≥ 50 years and NLR ≥ 3.13 are more likely to develop critical disease^15^. The counts of CD4+ cells and CD8+ cells in most patients in this group decrease significantly, but the specific mechanisms of lymphocyte subsets in such diseases needs to be further studied. More than 60% of patients have elevated levels of CRP, LDH and lactic acid, which may be related to the systemic immune response induced by infection and early hypoxia in critical patients. However, the correlation between these indicators and death and their predictive value for death risk need to be further investigated. Consistent with other studies, 77% of patients have increased D-dimer levels and are considered to be in a hypercoagulable state. Furthermore, the risk of thrombosis is increased because of long-term bed rest required by patients with severe disease. Thus, anticoagulant therapy should be actively given. Compared with young- and middle-aged patients who died, elderly decedents are more prone to myocardial injury, which may be related to the fact that elderly patients are more likely to have heart diseases.

ARF is the main cause of death in this group of patients who died, suggesting that SARS-CoV-2 infection mainly affects the respiratory system. Viral interaction with the angiotensin converting enzyme 2 (ACE2) receptor can cause systemic vascular endothelial injury, result in a hypercoagulable state, and cause thrombosis^16,17^. Most patients in this study had increased D-dimer levels, which also suggests that thrombosis may occur. However, due to the lack of a corresponding examination, the results are unavailable. Recently published autopsy reports^18^ on 12 cases of COVID-19 in Germany showed that four patients died of pulmonary embolism and three patients had venous thrombosis, suggesting that pulmonary embolism may be a cause of death in patients with ARF.

A number of studies have reported that coronavirus invades tissues and organs through the ACE2 receptor, which is abundant in the kidney and myocardium; moreover, autopsy results also confirmed that coronavirus can invade myocardial cells^19,20^. In this study, myocardial injury and acute kidney injury occurred in some patients who died. Whether the injury is caused by SARS-CoV-2 invasion or secondary changes caused by hypoxia is still uncertain. However, it can be determined that myocardial injury and acute kidney injury are not the main causes of death. At present, there are no antiviral drugs, including Kaletra^21^, that are effective for the treatment of COVID-19. Therefore, clinicians should focus on correcting hypoxemia caused by ARDS, maintaining stable vital signs and waiting for the body to eliminate the virus. It has been reported that the proportion of patients requiring mechanical ventilation of critical COVID-19 patients is 30:37^10^. Due to the shortage of medical resources at the time, 40.3% of the patients in this group received mechanical ventilation, and only 9.1% received endotracheal intubation. The time from when patients required endotracheal intubation to when they received it was long enough that some patients could not recover from ARF and eventually died from a lack of oxygen. There are reports that the incidence of pneumothorax in COVID-19 patients is approximately 1–2%, and the incidence in our study reached approximately 2.6%^6,11^, which is related to the fact that all the patients in this group were severely or critically ill. The aforementioned patient rapidly developed ARF and bilateral spontaneous pneumothorax after the onset of the disease. The cause of spontaneous pneumothorax in this patient may have been the large areas of consolidation in both lungs that resulted in decreased lung compliance, a very high respiratory drive, a vigorous inspiratory effort, and highly negative intrathoracic pressures, leading to pneumothorax^17^ and further aggravating the other manifestations of the disease. Therefore, appropriate respiratory support and adequate sedation and analgesia should be given to COVID-19 patients with early-stage ARF. In addition, clinicians should also be mindful of sepsis, the secondary cause of death in this study. Prior studies have shown that bacterial and fungal infections are common in the late stage of virus infection. A total of 30.3% and 16.9% of patients with severe H1N1 flu admitted to the ICU had bacterial or fungal infections, respectively. However, the incidence in COVID-19 patients is still unclear^11,22,23^. In this study, there were 7 patients with positive pathogen cultures, 2 of which were fungal pathogens. This result suggests that the incidence of fungal infection in COVID-19 patients may be lower than that of patients with severe H1N1 flu. However, due to the lack of alveolar lavage fluid and other lower respiratory samples, it is impossible to accurately evaluate the incidence of co-infection in COVID-19 patients. The occurrence of secondary bacterial and fungal infections in patients with COVID-19 needs further study.

## Conclusions

The majority of COVID-19 decedents are male, especially elderly decedents with underlying diseases. COVID-19 often starts with cough in female patients, and they are more likely than male COVID-19 patients to have diabetes. The proportion of young- and middle-aged decedents who were male is higher than that among elderly patients. Myocardial injury is common in elderly patients. Dyspnoea, along with a significantly decreased CD4+ cell count, is likely to occur in the early stage of COVID-19 in patients with hypertension. In the short-term survival group, mortality is not significantly associated with whether patients have hypertension; the majority of patients in the short-term survival group are still male and often have heart diseases, and the main cause of death is ARF. Of all 77 deaths, ARF and sepsis were the main causes of death.
